# ER-Phagy and Microbial Infection

**DOI:** 10.3389/fcell.2021.771353

**Published:** 2021-11-29

**Authors:** Jiahui Li, Enfeng Gao, Chenguang Xu, Hongna Wang, Yongjie Wei

**Affiliations:** ^1^ Affiliated Cancer Hospital and Institute of Guangzhou Medical University, Guangzhou, China; ^2^ Key Laboratory for Cell Homeostasis and Cancer Research of Guangdong Higher Education Institutes, Guangzhou, China; ^3^ GMU-GIBH Joint School of Life Sciences, Guangzhou Medical University, Guangzhou, China; ^4^ State Key Laboratory of Respiratory Disease, National Clinical Research Center for Respiratory Disease, Guangzhou Institute of Respiratory Health, Guangzhou, China

**Keywords:** ER-phagy, virus, infection, bacteria, microbial, reticulophagy, autophagy

## Abstract

The endoplasmic reticulum (ER) is an essential organelle in cells that synthesizes, folds and modifies membrane and secretory proteins. It has a crucial role in cell survival and growth, thus requiring strict control of its quality and homeostasis. Autophagy of the ER fragments, termed ER-phagy or reticulophagy, is an essential mechanism responsible for ER quality control. It transports stress-damaged ER fragments as cargo into the lysosome for degradation to eliminate unfolded or misfolded protein aggregates and membrane lipids. ER-phagy can also function as a host defense mechanism when pathogens infect cells, and its deficiency facilitates viral infection. This review briefly describes the process and regulatory mechanisms of ER-phagy, and its function in host anti-microbial defense during infection.

## Introduction

The endoplasmic reticulum (ER) is the largest membranous organelle in eukaryotic cells and consists of continuous lamellar and tubular structures spanning the cytoplasm. It is an essential intracellular reservoir for Ca^2+^ storage and a major site for protein synthesis, modification and transportation, as well as lipid and steroid synthesis (Schwarz and Blower, 2016). Correct folding of proteins by the ER involves a variety of mechanisms, and the process is essential for cell maintenance, proper function and growth (Shibata et al., 2006). Therefore, real-time quality control of the ER is critical to maintaining its homeostatic and appropriate operation and cell survival.

The compositions and structure of the ER are dynamic and sensitive to internal and external stimuli (e.g., ionizing radiation, chemicals, viruses, etc.). Any dramatic changes in the cell or environment can cause ER stress and damage, leading to loss of protein, impaired redox and calcium homeostasis, and worst of all, even cell death (Sprunger and Jackrel, 2019). Autophagy, a conserved cellular quality control mechanism to eliminate damaged organelles, plays an important role in maintaining ER health by degrading damaged ER fragments and the misfolded proteins encased within it that often cause these damages. ER-phagy typically targets the ER regions where aggregation-prone proteins are located and escorts them into autophagosomes for transport to vacuoles or lysosomes for degradation (Molinari, 2020). Recent studies have found that ER-phagy is also involved in the process of microbial infections, but its functions, regulatory mechanisms, and interplay with pathogens still need further investigation (Reggio et al., 2020). This article will review the current progresses, the molecular mechanisms of ER-phagy, and its interaction with pathogenic microorganisms when fighting infection. We hope our article will provide some insights for further exploiting ER-phagy as an anti-infection strategy.

## Overview of the ER

### Structure and Function of the ER

The ER is an essential organelle carrying protein synthesis, folding, assembly and transportation, as well as lipid metabolism, and is also the primary site for the storage of intracellular calcium (English and Voeltz, 2013). It consists of closed tubular or flattened vesicles and other encapsulated lumens, forming an interconnected three-dimensional lattice structure. The ER usually accounts for about half of the cell membrane and more than 10% of the total cell volume. Therefore, its presence dramatically increases the surface area of the inner cell membrane, providing a sizeable binding site for a variety of enzymes, especially for the multienzyme complexes. At the same time, the ER is an entirely closed membrane structure that separates the substances synthesized internally from the substances in the cytoplasm, which is more conducive to their processing and transportation (Hu et al., 2011).

Spatially, the ER starts from the outer membrane of the nucleus and extends outward to the entire cytoplasm. The ERs in the proximal nucleus region are generally lamellar in shape and relatively neatly arranged, with ribosomes distributed on them. Thus, the lamellar ER is a complex structure consisting of both ER membrane and ribosomes, whose primary function is to synthesize various secretory and membrane proteins. The network-like tubular ER is predominantly distributed in the proximal cell membrane region, spans the whole cytoplasms, and is the primary lipid synthesis site. They often serve as sites for outgrowth, and transfer proteins or lipids synthesized on the ER into the Golgi apparatus (Friedman and Voeltz, 2011).

A major function of the ER is the synthesis of membrane and luminal proteins, and about one-third of cellular proteins are synthesized on ribosomes attached to the ER membrane (Schwarz and Blower, 2016; Igbaria et al., 2019). In addition, the ER is the primary resident organelle for various post-translational modification enzymes and molecular chaperones. Thus, most newly generated polypeptides are transported into the ER for processes including shearing, post-translational modification, disulfide bond formation, and proper folding into mature proteins, which are subsequently sorted to different cellular sites to perform their respective functions (Benham, 2012; Daverkausen-Fischer and Pröls, 2021). Pathogens, such as viruses, typically hijack the host’s synthetic machinery to generate proteins for their replication and packaging. It is therefore reasonable to assume that the host ER is also a key organelle for pathogen reproduction (Inoue and Tsai, 2013; Chen et al., 2020).

### Quality Control of the ER

The size and shape of the ER are not fixed but dynamically changing, and its dynamics are critical for cellular homeostasis (Claessen et al., 2012). When the morphology and size of the ER become abnormal, the contact points of the ER with other organelles will change, and intracellular vesicle traffic and other ER functions will be affected. Under normal conditions, the half-life of ER membrane proteins and ER lipids is 3–5 days, but under stress conditions, the ER requires more active renewal to facilitate the quality control of itself and the proteins it produces (Chino and Mizushima, 2020).

The key to ER quality control is to prohibit the aggregation of unfolded proteins in the lumen (Wilkinson, 2020). Many harsh stimuli can disrupt the protein folding process in the ER, producing large amounts of misfolded and unfolded proteins that accumulate in the lumen that overburdens the ER and create a pathological state known as ER stress. Cells have evolved a complex mechanism called the unfolded protein responses (UPR) to sense and respond to ER stress and prevent damages. The UPR is initiated by activating at least three stress response sensors, including inositol-requiring protein 1 (IRE1), protein kinase RNA-like ER kinase (PERK) and activating transcription factor 6 (ATF6) (Bhattacharyya et al., 2014). All three sensor proteins contain ER transmembrane domains that facilitate their localization and transverse the ER membrane, with one side facing the cytoplasm and the other facing the ER lumen. They bind to unfolded proteins via domains within the ER lumen, initiating UPR signaling and subsequently stimulating molecular chaperones production to enhance protein folding and translocation, or stimulating ER-associated degradation (ERAD) to degrade proteins that are still not properly folded. ERAD is executed by the 26S proteasome complex, whose substrates include soluble and integral membrane proteins, polypeptide chains that have not completed post-translational modifications or are misfolded, and unassembled members of protein complexes (Ruggiano et al., 2014).

Not all misfolded proteins in the ER are suitable for degradation by ERAD, so autophagy needed to be involved in their clearance (Chino and Mizushima, 2020). Under physiological conditions, ER-phagy is kept at basal levels to maintain ER homeostasis; however, under adverse conditions such as nutritional deficiencies, ER stress, protein aggregation or pathogen invasion, ER-phagy is significantly elevated (Dikic, 2018). Previous studies have clarified that ER-phagy was involved in two complementary cellular processes, namely the degradation and the restoration of the stress-damaged ER (Anding and Baehrecke, 2017). ER-phagy promotes cell survival by degrading damaged ER fragments and their encased misfolded protein aggregates to release ER stress, and sometimes by degrading healthy ER when nutrients are deficient. When the stress from the unfolded protein response subsides, ER-phagy reduces the size of the ER and restores it to healthy proportions. The degradation and restoration functions of ER-phagy work in concert to ensure ER homeostasis and function (Loi et al., 2018). Conversely, defective ER-phagy will result in the accumulation of misfolded protein aggregates in the ER, triggering a burgeoning UPR response that promotes inflammation, cell death and even tumorigenesis (Grootjans et al., 2016).

## The Discovery History and Molecular Mechanism of ER-Phagy

### Overview of Autophagy and ER-Phagy

Autophagy is the process by which cells transport cytoplasmic cargo to lysosomes for degradation and reuse to maintain cyclic turnover and cellular energy requirements, which is essential for stress mitigation, homeostasis maintenance and cell differentiation. Autophagy is usually induced by stress factors such as hypoxia, energy or amino acid deficiency, radiation, drugs, and infection (Levine and Kroemer, 2019). After autophagy is initiated, some double-membrane structured phagophores start to form and wrap around the cytoplasmic substances to be degraded and gradually expand and close to create autophagosomes, which then fuse with lysosomes to form autophagolysosomes. In the presence of an acidic environment and lysosomal enzymes, substances encapsulated inside autophagolysosome are degraded into amino acids, nucleotides, and free fatty acids to be reused to synthesize macromolecules or generate energy. The autophagy marker microtubule-associated protein one light chain 3 (LC3) in a phosphatidylethanolamine-conjugated form (LC3-PE) localizes to the inner and outer membranes of autophagic vesicles formed at each stage of the autophagic process and mediates the fusion and aggregation of lipid membranes (Klionsky et al., 2016).

Autophagy can both non-selectively degrade various intracellular components and selectively target damaged organelles and cellular structures for degradation, the latter process being referred to as selective autophagy (Li et al., 2021). The specificity of selective autophagy is determined by the specific interaction between LC3-PE and the selective autophagy receptors (SAR), which contain a [W/F/Y]xx [L/I/V]) tetrapeptide sequence (Chino et al., 2019). Depending on the degradation substrate, selective autophagy can be further classified into lipophagy for degrading liposomes, peroxiphagy for degrading peroxisomes, ribophagy for degrading ribosomes, mitophagy for degrading mitochondria and ER-phagy for degrading ER, etc (Jin et al., 2013).

**FIGURE 1:**
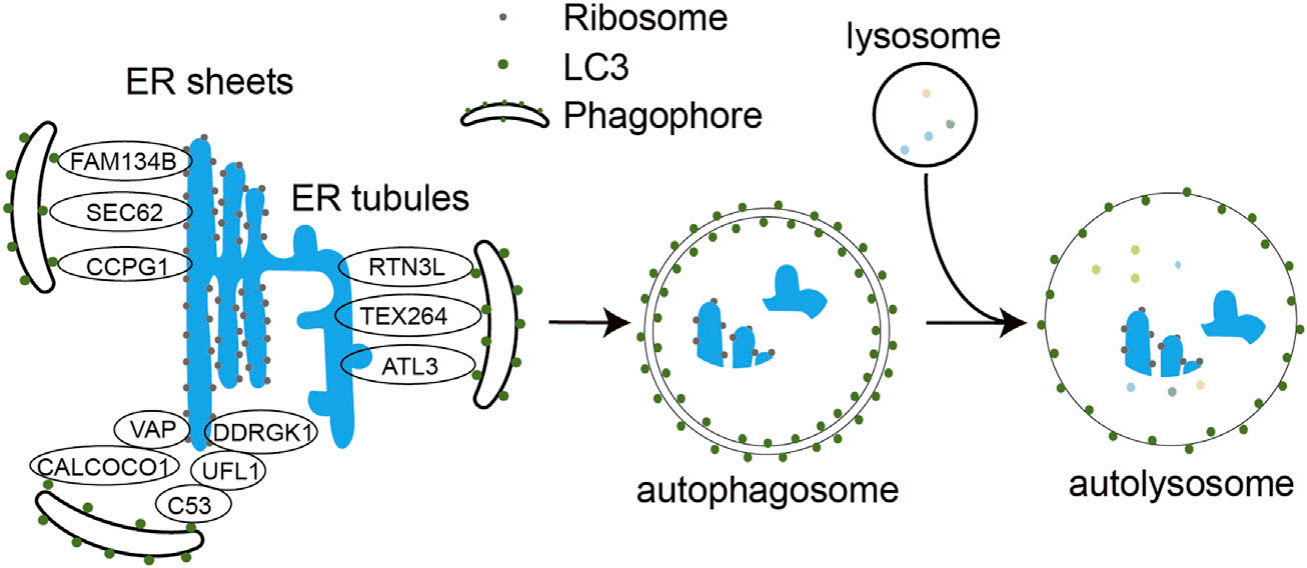
Molecular mechanism of ER-phagy. Under different stress conditions, ER-phagy receptors FAM134B, SEC62, RTN3L, ATL3, TEX264, CCPG1, C53, and CALCOCO1 bind to LC3 via their LIR motifs and engulf ER structures in need of degradation into the phagocytic vesicles, which subsequently expand and close to form double-membrane autophagosomes. The autophagosome further fuses with the lysosome to form autophagolysosome. The encapsulated ER fragments are eventually degraded by hydrolases in the autolysosomes.

ER-phagy is one of the most critical quality control mechanisms for the ER, usually activated when incorrectly folded proteins are not cleared in time and accumulate thus leading to ER stress and ER damage (Chino and Mizushima, 2020). Upon activation, it targets these damaged ER fragments to the lysosome for removal by the autophagy machinery. Based on the different processes by which ER fragments are wrapped and transported to the lysosome, ER-phagy is divided into three forms: macro-ER-phagy, micro-ER-phagy, and LC3/Atg8-dependent vesicle delivery pathway (Molinari, 2021). Macro-ER-phagy uses autophagosomes to encapsulate isolated ER fragments, transport them and fuse them with lysosomes for degradation (Figure 1). It is usually used to deal with the nutrient shortage, ER stress, ribosome stalling, and accumulation of polypeptide chains. In micro-ER-phagy, the ER fragments are directly engulfed by lysosomes or late endosomes. In the LC3/Atg8-dependent vesicle delivery pathway, vesicles containing unfolded or misfolded proteins bud from the ER membrane and directly fuse with lysosomes. Its primary function is to remove misfolded proteins in the ER (Chino and Mizushima, 2020). Of the three ER-phagy modalities, macro-ER-phagy has received the most attention and is best characterized. The infection-associated ER-phagy discussed in the latter sections of this review all refers to macro-ER-phagy.

### The Discovery History of ER-Phagy

In 1973, Bolender and Weibel used transmission electron microscopy to measure a 26% increase in hepatocyte volume in the rat after five consecutive days of intraperitoneal administration of sodium phenobarbital (100 mg/kg dissolved in saline). Growth of the cytoplasmic matrix was the leading cause of the hepatocyte swelling, with a one-fold expansion in volume and 90% increase in surface area of the smooth ER. Five days after stopping the drug, the enlargement of the smooth ER disappeared, along with a dramatic 8-fold increase in the volume and 96% increase in the number of autophagic vesicles. Morphological analysis of the autophagic vesicles showed that their components were mainly the disappearing ER membranes. This result suggests that the formation of autophagic vesicles is not random and that the increased autophagic activity is associated with the selective removal of excess ER membranes (Bolender and Weibel, 1973). In 2006, Bernales et al. found that administration of dithiothreitol (DTT), which disrupts protein disulfide bonds, and clathrin, which interferes with N-terminal glycosylation of proteins, significantly induced ER stress in yeast. DTT treatment results in a rapid expansion of the ER membrane of yeast cells and the appearance of a large amount of double-membrane-encapsulated, 300- to 700-nm- diameter vesicles observable under the electron microscope. These vesicles contain densely packed membrane structures with a dense distribution of ribosomes on the outer membrane and the extension, indicating that they originate from ER. Further immunogold staining of the ER marker protein SEC61 also supported the conclusion that the vesicular inclusions were of ER origin. Therefore, Bernales et al. named these vesicles ER-containing autophagosomes (ERAs) (Bernales et al., 2006). The near absence of cytoplasm and other organelles within ERAs suggests that these ER membranes are selectively phagocytosed. In the same year, Ogata et al. also found that neuroblastoma SK-N-SH cells administered with 2 μg/ml clathrin for 2 h or 1 μM toxic carotene for 6 h showed autophagosomes containing multilamellar structures of the ER (Ogata et al., 2006). Based on these findings, Bernales et al. first proposed the concept of ER-phagy as organelle-selective macroautophagy induced by ER stress in 2007 (Bernales et al., 2007).

It is worth noting that the above pathways are species-specific in yeast and mammals, and their conservation has yet to be explored. The existence of ER-phagy was further clarified in 2015 when two research teams, Nakatogawa and Dikic, reported the first ER-phagy receptor Atg40 in yeast and the mammalian counterpart FAM134B, respectively, at the same time (Khaminets et al., 2015; Mochida et al., 2015). With the discovery of additional ER-phagy receptors, the understanding of the molecular mechanisms of ER-phagy in eukaryotic cells and the associated diseases has been further accelerated.

### ER-Phagy Receptors in Mammalian Cells

ER-phagy links the ER fraction to be degraded to the autophagic machinery via ER-phagy receptors. In yeast, the ER-phagy receptors bind to Atg8 on autophagosomes through the Atg8-interacting motif (AIM), facilitating the encapsulation of the ER by the autophagosome. There is only one ATG8 in yeast; however, six Atg8 homologs have been identified in mammals, including three isoforms of LC3 (LC3A, LC3B, and LC3C) and three isoforms of γ-aminobutyric acid (GABA) type-A receptor-associated protein (GABARAP, GABARAPL1, and GABARAPL2). As with the interaction between ER-phagy receptors and ATG8 in yeast, mammalian ER-phagy receptors also bind specifically to the LC3-interaction region (LIR) motif of these ATG8 homologs to facilitate the encapsulation of the ER fraction by the autophagosome (Chino and Mizushima, 2020).

Eight mammalian ER-phagy receptors have been identified, including six transmembrane proteins (FAM134B, Sec62, RTN3 long isoform (RTN3L), CCPG1, ATL3 and TEX264) and two soluble proteins (CALCOCO1 and C53) (Figure 1). FAM134B, a member of the FAM134 protein family, contains a LIR motif capable of binding both LC3 and GABARAP at the carboxyl terminus, is the most studied ER-phagy receptor (Khaminets et al., 2015). Its transmembrane reticulon-homology domain (RHD) contains two wedge-shaped transmembrane helix clamps and two amphipathic helix structures that form two hydrophobic wedges on the outside of the phospholipid bilayer. This structure facilitates ER membrane bending, which allows FAM134B to interact with LC3/GABARAP via the cytoplasmic LIR motif and functions as an ER-phagy receptor. Reticulons (RTNs) are membrane proteins that are highly enriched in the tubular ER and are associated with ER remodeling. Four subfamilies of RTNs have been identified to date, and each subfamily has many different spicing isoforms. Among all these isoforms, only the longest RTN3L has the function of ER-phagy receptor. Under amino acid starvation, RTN3L overexpression induces fragmentation of tubular ERs containing RTN1, RTN3, and RTN4 and mediates their macro-ER-phagy (Grumati et al., 2017). ATL3 is mainly localized to the highly curved ER membrane, including the edges of the tubular ER and lamellar ER. ATL3 contains a GTPase domain, an RHD domain and two GABARAP interaction motifs (GIMs) that interact with the GABARAP subfamily of proteins, which are required for ATL3 to function as a tubular ER-phagy receptor. In amino acid-starved COS-7 cells, ATL3 dominates macro-ER-phagy (Liang et al., 2018). Testis expressed gene 264 (TEX264) is mainly localized to the three-way junctions of the ER, and it is a transmembrane protein with a cytoplasmic domain containing LIR. TEX264 is responsible for more than half of the starvation induced ERphagic flow in the cell. However, not all ER proteins are responsive to TEX264-mediated ER-phagy, and proteomic studies also confirm that TEX264 may only target specific ER regions (Chino et al., 2019). SEC62 is a component of the translocon complex SEC61/SEC62/SEC63 and is involved in the post-translational insertion of nascent peptides into the ER. SEC62 mediates macro-ER-phagy and micro-ER-phagy to regulate ER turnover, mainly during recovery from acute ER stress (Fumagalli et al., 2016). CCPG1 is a vertebrate-specific protein that is induced during ER stress and activates peripheral ER autophagy. The transmembrane domain of CCPG1 anchors it to the ER membrane and contains a LIR motif in the cytoplasmic tail (Smith et al., 2018).

C53, a tumor suppressor in mammals, was identified as the first cytosolic ER-phagy receptor. It contains three non-canonical AIMs termed shuffled AIM (sAIM) that interact with ATG8 family proteins. In response to proteotoxic stresses in the ER lumen, C53 interacts with the UFMylation E3 ligase UFL1 and its ER membrane adaptor protein DDRGK1 to form a heteromeric receptor complex for ER-phagy. Stalled ribosome activates the C53/UFL1/DDRGK1 receptor complex to trigger the autophagic degradation of internal and passenger proteins in the ER (Stephani et al., 2020). CALCOCO1 is another soluble ER-phagy receptor involved in the degradation of tubular ER induced by toxic proteins or starvation. It contains an FFAT (two phenylalanines (FF) in an acidic tract)-like motif and a UIM (ubiquitin interacting motif) domain at the carboxyl terminus and a LIR domain at the amino terminus. CALCOCO1-mediated ER-phagy requires that CALCOCO1 interacts with the ER membrane protein VAP through the FFAT-like structural domain. Knockdown of CALCOCO1 results in ER expansion (Nthiga et al., 2020). Besides the receptors mentioned above, p62 protein has also been reported to mediate ER-phagy to degrade excess ER generated by exogenous chemical stimulation in rat liver (Yang et al., 2016).

## ER-Phagy and Infection

### Viral Infection and ER-Phagy

As strictly intracellular parasitic organisms, viruses “hijack” various cellular processes and actively regulate or alter the intracellular environment to create favorable conditions for their replication. The ER is often a favored organelle for viruses to hijack due to the presence of a variety of resident enzymes, chaperones and receptors, and its expanded membrane structure and connection to other organelles (Bagchi, 2020). Once a virus that relies on the ER for replication establishes an effective infection, the exponential replication of viral particles is accompanied by the rapid aggregation of large amounts of viral proteins in the ER, which also coincides with the synthesis requirements of host cell proteins (Reggio et al., 2020). Overloaded protein processing in the ER will likely lead to a cellular response to viral infection by initiating UPR-mediated protein degradation or ER-phagy to inhibit viral protein synthesis, thereby preventing viral replication. On the other hand, viruses have also evolved mechanisms to manipulate UPR and ER-phagy, thereby creating a favorable environment for their proliferation and sustained infection (Chen et al., 2020; Reggio et al., 2020).

ER-phagy has been reported to assist host resistance to infection and limit the replication of Ebola, dengue and Zika viruses (ZIKVs) (Table 1). Ebola virus is a virulent pathogen that causes severe Ebola hemorrhagic fever (EHF) in humans and primates with a high mortality rate (Holmes et al., 2016). Mouse embryonic fibroblasts (MEFs) from Fam134b knockout mice infected with the Ebola virus showed a 10- to 100-fold increase in viral yield and an elevated expression of viral GP, VP40, and nucleocapsid proteins, indicating that Fam134b-dependent ER-phagy restricts the virus replication (Chiramel et al., 2016). The Ebola virus glycoprotein (GP) protein is a trimeric glycoprotein on the surface of virion particles, playing a pivotal role in the assembly, maturation, and virulence of the virus. It undergoes glycosylation and shearing in the ER before being transferred to the Golgi apparatus to become the mature GP1 and GP2 proteins (Volchkov et al., 1998; Lennemann et al., 2014; Yu et al., 2017). Overexpressed full-length GP accumulates in the lamellar ER and causes cytotoxicity (Bhattacharyya and Hope, 2011). If and how Ebola virus triggers ER-phagy in host cells are yet to be determined. However, it is conceivable that viral infection requires a burst of GP synthesis, thus causing GP accumulation in the ER and ER stress, and is a likely trigger for ER-phagy. Therefore, selectively degrading ER fragments containing viral proteins by ER-phagy is a reasonable coping strategy to limit viral replication (Chiramel et al., 2016). However, the precise mechanism by which Fam134b knockout leads to the failure of restriction on Ebola virus replication remains to be investigated. And the study will be beneficial in establishing animal models and preclinical anti-infection strategies for the Ebola virus.

**TABLE 1:** The regulatory role of ER-phagy in infection.

Microbial pathogens	Experimental cell type	Pathogen effector	ER-regulator	ER-phagy functions	References
Ebola virus	Mouse embryonic fibroblast	ND	Fam134b	Suppresses Ebola virus proliferation	Chiramel et al. (2016)
DENV, ZIKV	Human brain microvascular endothelial cells	NS3	FAM134B	Inhibits viral replication	Lennemann and Coyne, (2017)
DENV, ZIKV	Human alveolar basal epithelial cells	Dengue viral NS1, capsid and Env	ATL2, ATL3	ND	Monel et al. (2019)
WNV					
DENV, ZIKV	Human brain microvascular endothelial cells	ND	FAM134B	BPIFB3 enhances FAM134B-dependent ER-phagy, restricts viral replication	Evans et al. (2020)
FMDV	pig kidney cell line	ND	Sec62	Promotes ER-stress restoration, inhibits viral replication	Wu et al. (2021)
Gram-positive infectious bacteria	Human macrophage	c-di-AMP	STING	Resolves ER stress and rescues phagocytes from death upon infection, controls the enhanced interferon response to infectious live Gram-positive bacteria	Moretti et al. (2017)
*Mycobacterium tuberculosis*	Murine macrophage	ND	P62	Bag2 induced ER-phagy mediates antibacterial defense in macrophages, mitigates ER stress and protects macrophages from apoptosis	Liang et al. (2020)

ND, not determined.

**FIGURE 2:**
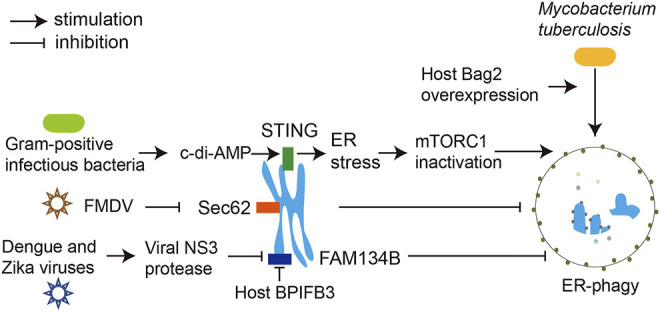
Interaction of pathogenic microorganisms with ER-phagy. DENVs and ZIKVs inhibit FAM134B-mediated ER-phagy through their NS3 protease, preventing ER autophagic degradation and thus ensuring the availability of synthetic building blocks from the ER necessary for their replication. Host BPIFB3 inhibits FAM134B-dependent ER-phagy in flavivirus-infected cells. For FMDVs, their infection inhibits Sec62-mediated ER-phagy. When Gram-positive bacteria infect cells, the c-di-AMP produced activates the ER-localized immune sensor STING, which subsequently inhibits mTORC1 and initiates ER-phagy to fight the infection. Of note, overexpression of host factor Bag2 in the *Mycobacterium tuberculosis-*infected murine macrophages induces ER-phagy.

Flaviviruses are a large group of single positive-stranded RNA viruses with an envelope. Most of the flaviviruses are important human pathogens, such as Zika virus (ZIKV), West Nile virus (WNV), dengue virus (DENV), yellow fever virus (YFV), and Japanese encephalitis virus (JEV). Although some flavivirus infections have no obvious symptoms or only mild symptoms, severe flavivirus infections can lead to hemorrhagic fever, viral encephalitis, meningitis, biphasic fever, delayed paralysis, jaundice, and various neurological complications, and some flaviviruses persist in the patient’s body and cause long-term disease (Pierson and Diamond, 2020). Flaviviruses are closely associated with ER, and DENV, ZIKV, and WNV can all use ER as a source of membranes to replicate in the host (Monel et al., 2019; Neufeldt et al., 2019). Knockdown of FAM134B with siRNA in host cells resulted in enhanced replication of DENVs and ZIKVs and a more than 10-fold increase in the cells’ viral output. This result suggests that the host cells also use FAM134B-mediated ER-phagy to limit viral access to resources from the ER and prevent their amplification. Meanwhile, DENVs and ZIKVs have developed a strategy to antagonize ER-phagy using their NS3 protease to cleave the RHD domain of FAM134B, thus preventing its oligomerization and ER-phagy receptor function and impeding the formation of ER- and viral protein-containing autophagosomes (Lennemann and Coyne, 2017) (Figure 2). Therefore, the specific protease inhibitor of viral NS3 may be a promising anti-flavivirus drug worthy of immediate development. BPIFB3 (bactericidal/permeability-increasing protein (BPI) fold-containing family B, member 3) is an ER-localized host protein belonging to the antimicrobial proteins of the BPI superfamily (Balakrishnan et al., 2013; Morosky et al., 2016; Evans et al., 2021). Recent studies have shown that its knockdown enhances FAM134B-dependent ER-phagy in cells infected with DENVs and ZIKVs, increases ER degradation, reduces viral acquisition of ER membranes for replication, and prevents viruses survival. Simultaneous knockdown of BPIFB3 and FAM134B reversed the ER-phagy enhancement triggered by BPIFB3 knockdown, allowing DENVs and ZIKVs to recover their replication capacity. This result indicates that DENVs and ZIKVs exploit host BPIFB3 to inhibit ER-phagy in host cells to promote their own reproduction, but the exact mechanism remains to be further investigated (Evans et al., 2020). How the NS3-mediated and BPIFB3-mediated ER-phagy inhibition pathways interplay remains unknown, but the observation that viruses exploit both their own encoded and host proteins against the exact antiviral mechanism in the host cells provides new insights into the virus-host interactions. In contrast to FAM134B, the ER-phagy receptors RTN3 and ATL3 of tubular ER appear to play opposite roles for flavivirus proliferation. Knockdown of host RTN3 or ATL3 significantly affected replication of DENVs, ZIKVs and WNVs and reduced the output of these viruses (Aktepe et al., 2017; Neufeldt et al., 2019). This manifestation may be attributed to the direct involvement of ATL3 in virus assembly and RTN3 in viral replication (Aktepe et al., 2017; Neufeldt et al., 2019). Meanwhile, whether RTN3 and ATL3 are responsible for flavivirus infection-induced tubular ER autophagic turnover and whether tubular ER autophagy is involved in virus multiplication still needs further investigation.

Foot-and-mouth disease virus (FMDV) is a small RNA virus that infects even-toed ungulates and causes high fever, characteristic blisters, hemorrhagic gastroenteritis and myocarditis in young animals (Berryman et al., 2012). In FMDV-infected porcine kidney PK-15 cells, Sec62/LC3 interactions and co-localization of Sec62, LC3 and virus were detected, suggesting that Sec62-mediated ER-phagy is involved in the FDMV propagation (Table 1). Sec62 knockdown led to elevated viral production, while overexpression of Sec62 inhibited viral replication by enhancing viral infection-triggered ER-phagy (Wu et al., 2021). All these indicate an important regulatory role of Sec62-mediated ER-phagy in FDMV infection and its potential as a target for antiviral intervention.

### Bacterial Infection and ER-Phagy

For intracellular bacteria, it is essential to use the organelles and molecular mechanisms of the host cell to generate a microenvironment suitable for their survival and proliferation. The ER is a nutrient-rich intracellular site for bacteria without anti-microbial peptides and hydrolytic enzymes, making it an amicable environment for bacterial intracellular survival (Roy et al., 2006; Celli and Tsolis, 2015). Electron microscopic observations that *Legionella pneumophila* and *Brucella spp.* grow in specialized vacuoles filled with rough ER with ribosomes attached, demonstrating interactions between bacteria and ER. *Legionella longbeachae*, *Chlamydia trachomatis*, and the *chlamydiosis-associated Simkania negevensis* have also been observed to replicate in organelles adjacent to the ER (Celli and Tsolis, 2015). Correspondingly, hosts have developed ER-associated response mechanisms to defend against bacterial infection.

STING is an ER membrane protein containing four transmembrane helices and is a crucial regulator of innate immunity, the first line of defense against invading pathogens. Pathogen-derived DNA recognizes and activates the intracellular DNA receptor cyclic GMP-AMP synthase (cGAS), which catalyzes the synthesis of cGAMP from ATP and GTP. cGAMP acts as a second messenger of STING, driving STING conformational changes and self-activation. Subsequently, TBK1, a critical kinase to innate immune responses, is recruited to STING and activated to phosphorylate the downstream transcription factor interferon regulatory factor 3 (IRF3), which transcribes innate immune cytokines (Ishikawa and Barber, 2008; Ishikawa et al., 2009). When Gram-positive bacteria such as *Listeria monocytogenes* infect macrophages, STING senses c-di-AMP produced by internalized bacteria and rapidly manipulates downstream cellular responses, including ER stress, interferon responses, mTOR inactivation, and ER-phagy (Figure 2) (Moretti et al., 2017). ER-phagy is thought to dissolve ER fragments that are severely stressed by infection, thereby rescuing macrophages from death, but the ER-phagy receptors involved in this process have not been identified (Dikic, 2018).

*Mycobacterium tuberculosis* (M.*tb*), the causative agent of tuberculosis, can attack all body organs and lead to various diseases, of which pulmonary tuberculosis is the most common and still prevalent today (Koch and Mizrahi, 2018). M.*tb* infection induces ER-phagy in macrophages. Overexpression of Bcl-2-associated athanogene 2 (Bag2), an HSP70 co-chaperone protein involved in the pathogenesis of various diseases, further elevated the ER-phagy level. Further studies showed that p62 was more concentrated on the ER in Bag2 overexpressing macrophages, suggesting that p62 is involved in Bag2-mediated ER-phagy (Table 1). This ER-phagy was proposed to be the mechanism that eliminates ER stress from M.tb infection and prevents apoptosis of macrophages (Liang et al., 2020).

## Concluding Remarks

The continued discovery of multiple types of selective autophagy and their interactions with pathogenic factors at different steps of the pathogen life cycles have broadened our knowledge of how pathogens and hosts antagonize and coexist with each other. The discovery of ER-phagy has further enriched the understanding of selective macroautophagy and organelle autophagy, expanding and deepening the study of ER stress and subsequent pathways mediated by the UPR. The current identified ER-phagy receptors and the pending discovery of novel receptors in response to different stimuli provide the basis for in-depth study of the mechanism of ER-phagy and its role in resistance to pathogenic microbial invasion.

Previous studies on infection-induced ER-phagy suggest that further research on this topic will provide new insights and entry points for understanding the mechanisms and developmental processes of infectious diseases and developing novel methods of prevention and treatment. FAM134B-mediated ER autophagy effectively limits viral replication and is, therefore, a potential target for antiviral intervention. However, there are significant limitations in understanding the relationship between ER-phagy and viral or bacterial replication in the host because these studies are based on *in vitro* cell-culture rather than *in vivo* infection models. Therefore, further studies on ER-phagy in patients with chronic viral infections are of great scientific and clinical value. In addition, the physiological function of ER-phagy, the regulatory mechanism, and the relationship with more diverse infectious diseases still need further investigation, and these studies are expected to lead to novel strategies for manipulating ER-phagy to fight infection.
